# Supplementary material for: Flexible employment policies, temporal control and health promoting practices: A qualitative study in two Australian worksites
Source: PLoS One. 2019 Dec 20;14(12):e0224542. doi: 10.1371/journal.pone.0224542 (PMC6924681; doi:10.1371/journal.pone.0224542)
Supplement: S1 File — (DOCX) [file pone.0224542.s001.docx]

**S1 File. Employer Recruitment Letter**

**Work, Time and Health Study**

The *WORK, TIME AND HEALTH STUDY* is investigating the relationship between working time arrangements and healthy lifestyles. This two year study is being conducted by the National Centre for Epidemiology and Population Health at the Australian National University, funded by the Australian Research Council.

This information sheet provides background information on the study, how your organisation can be involved and what participants will be asked to do.

**What is this research about?**

Over the last 30 years, Australian workplaces have changed and the ‘Monday-to-Friday, nine-to-five’ model of work is no longer the norm. Globalisation, new technologies and the demand for convenience by consumers means that workplaces operate much more flexibly than in the past. At the same time, more women and young people have entered the workforce and older workers are staying longer before retirement, driving demand for more flexible working arrangements, such as part-time work, flexitime or work from home.

Many workplaces now offer flexible arrangements to help their workers balance paid work with other responsibilities including family, study or community involvement. However, little is known about the potential impacts on, or benefits of, these changes for employee health. Understanding the impacts of changing workplaces and patterns of work for health is important, because the health of our workforce has important flow-on effects for business and economic growth. For example, healthy workers take less sick-leave, have fewer workplace accidents, are more likely to be more productive while at work, and may be able to extend their working lives.

The aim of this research is to find out about how flexible working arrangements affect people’s capacity to carry out daily activities that influence their health, such as physical activity, healthy eating and maintaining relationships with family members and friends.

To investigate these issues, the researchers will be collecting information on people’s working arrangements, along with details about their non-work activities. The aim is to see how they see these two aspects of their lives affecting one another. From this, we will be able to shed light on how the changing nature of work is affecting people’s health and productivity.

The study has three phases:

Phase 1 – Analysis of existing national survey data to identify emerging patterns of work-time organisation amongst Australian employees and the impact of different work-time arrangements on health.

Phase 2 – Interviews with employees across a range of work-time conditions to explore the impact of work on health promoting activities (e.g. healthy eating, physical activity).

Phase 3 – Interviews with stakeholders to identify what role health considerations might play in future debates about working time flexibility.

**How can we be involved?**

The research team is looking for a number of employers to participate in the study by helping our research team identify a small number of their employees to take part in interviews. We are hoping to recruit 60 employees in total from across a number of organisations.

**What will employers and employees be asked to do?**

Employers

Employers will be asked to help the researchers to identify suitable employees to take part in the research. We are interested in interviewing employees across a range of working conditions (i.e. full-time, part-time, permanent, casual, shift work). We are happy to be guided by employers in determining the best way to identify potential participants through, for example, working with Human Resources. Employers will not be named in any work which follows from the study.

Employees

Employees will be asked to keep a brief 24 hour ‘time diary’ to record their work and non-work activities. Once completed, they will be asked to make a time to talk with the research team.

This formal interview will take around 45 minutes and can be conducted at a time and place convenient to both employers and employees (i.e. before or after work).

In the interview, employees will be asked about their experiences of fitting work and other responsibilities (e.g. child care, healthy lifestyles) into their day-to-day lives. Employees will also be asked some brief questions about their occupation, hours and personal circumstances (e.g. structure of their household). This information will be confidential and employees will not be named in any work arising from the study.

**What do we get in return?**

At the conclusion of the research a short report will be provided to your organisation. The report will outline how flexible working arrangements are helping your employees to maintain healthy lifestyles and identify potential areas for improvement that, in turn, could boost the productivity of your workplace by improving the health of your workers.

The research team would also be happy to come and give a presentation on the findings to you and/or your staff.

**Research team:** Associate Professors Jane Dixon, Lyndall Strazdins, Cathy Banwell, Lara Corr & Drs Gemma Carey, Danielle Venn and Ginny Sargent (Australian National University); Dr Dan Woodman (University of Melbourne); Professor John Burgess (Curtin University); and Professor Michael Bittman (University of New England).
