# Supplementary material for: Flexible employment policies, temporal control and health promoting practices: A qualitative study in two Australian worksites
Source: PLoS One. 2019 Dec 20;14(12):e0224542. doi: 10.1371/journal.pone.0224542 (PMC6924681; doi:10.1371/journal.pone.0224542)
Supplement: S2 File — (DOCX) [file pone.0224542.s002.docx]

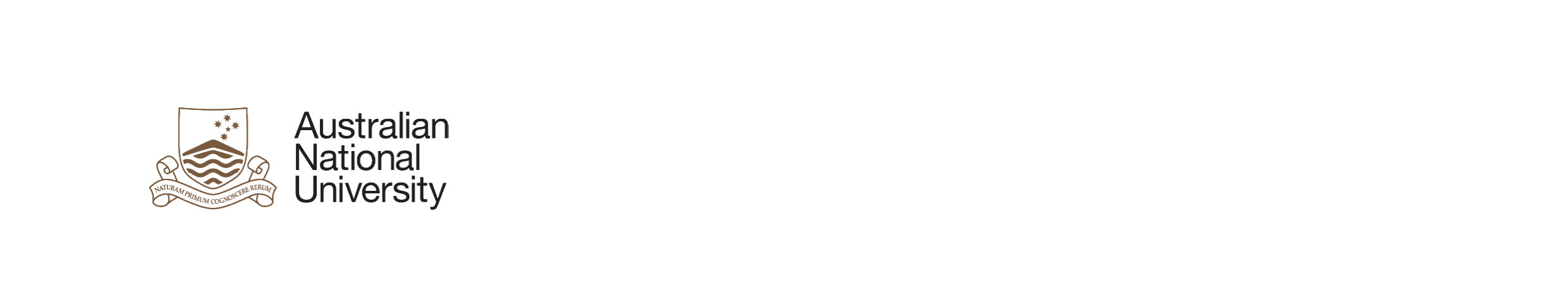
**National Centre for Epidemiology and Population Health**

**College of Medicine, Biology and Environment**

Canberra ACT 0200 Australia

**S2 File. Employee Information Letter**

Dear Sir/Madam,

**RE: Work, Time & Health Study**

We invite you to take part in a study entitled *Work, Time & Health.*  The Project is funded by the Australian Research Council (ARC). The study is being run by the National Centre in Epidemiology and Population Health, at the Australian National University, by (Chief Investigation) Dr Jane Dixon.

The aim of this research is to find out about how working hours affect people’s capacity to carry out daily activities, such as physical activity healthy eating and relationships with family members.

To find out about these issues, the researchers are collecting information on people’s work hours, non-work activities and how they see these two aspects of their lives affecting one another. From this, we will be able to shed light on how deregulation and workplace flexibility is affecting people’s health.

If you choose to participate, you will be asked to keep a short ‘time diary’ of your work and non-work activities for two 24 hour periods (one Sunday, and one weekday). After completing the diary, you will then be invited to take part in an interview to discuss their work and non-work hours and activities in more detail.

In the next few days, a representative of the project team will contact you to ask whether you are interested in participating. If you are, they will arrange for a copy of the time diary to be sent to you, after which a date and time for the interview will be organised. The time diary will be accompanied by a consent and information sheet. We will collect a copy of the consent form when we meet for the interview, along with the completed time diary.

All information collected will be anonymous. Involvement in the project is voluntary and you are free to withdraw consent at any time, and to withdraw any unprocessed data previously supplied to the researcher.

Sincerely,

Dr Jane Dixon (Chief Investigator) & Lara Corr (Research Fellow)

On behalf of the ‘Work, Time and Health’ project team.
