# Supplementary material for: Flexible employment policies, temporal control and health promoting practices: A qualitative study in two Australian worksites
Source: PLoS One. 2019 Dec 20;14(12):e0224542. doi: 10.1371/journal.pone.0224542 (PMC6924681; doi:10.1371/journal.pone.0224542)
Supplement: S3 File — (DOCX) [file pone.0224542.s003.docx]

**S3 File. Consent Protocol**

**WRITTEN CONSENT** for Participants **WORK, TIME & HEALTH STUDY**

I have read and understood the Information sheet you have given me about the research project and I agree to participate in the project and understand that I can withdraw from the research at any time.

Signature:…………………………………………….

YES ☐ NO  I agree to this interview being audio taped

YES ☐ NO  agree to be identified through a pseudonym

YES ☐ NO  I agree to provide the researchers with a time diary

Signature:…………………………………………….

**IF you would like to receive a summary of the research once it is completed**, please supply contact details (otherwise leave blank)**.**

Postal address:

**OR** Email address:
