# Supplementary figures and images for: Flexible employment policies, temporal control and health promoting practices: A qualitative study in two Australian worksites
Source: PLoS One. 2019 Dec 20;14(12):e0224542. doi: 10.1371/journal.pone.0224542 (PMC6924681; doi:10.1371/journal.pone.0224542)

**S4 File. Weekday Time Diary**


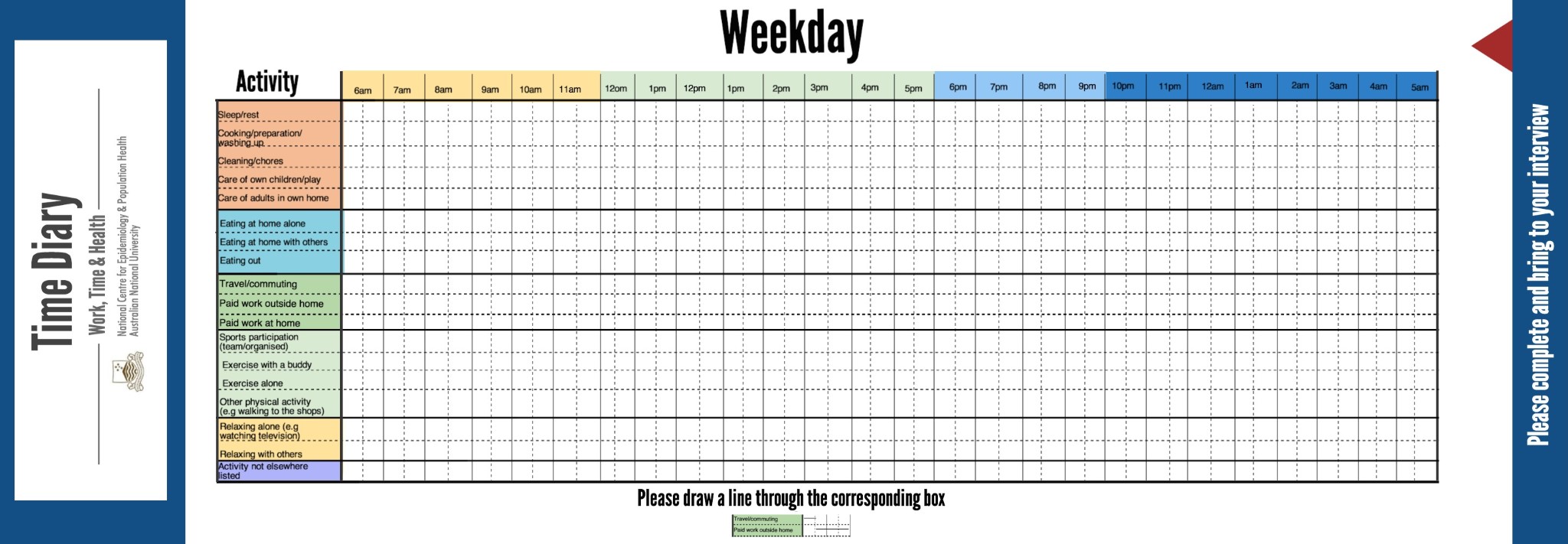

Supplement: S4 File — (DOCX) [file pone.0224542.s004.docx]

**S5 File. Sunday Time Diary**


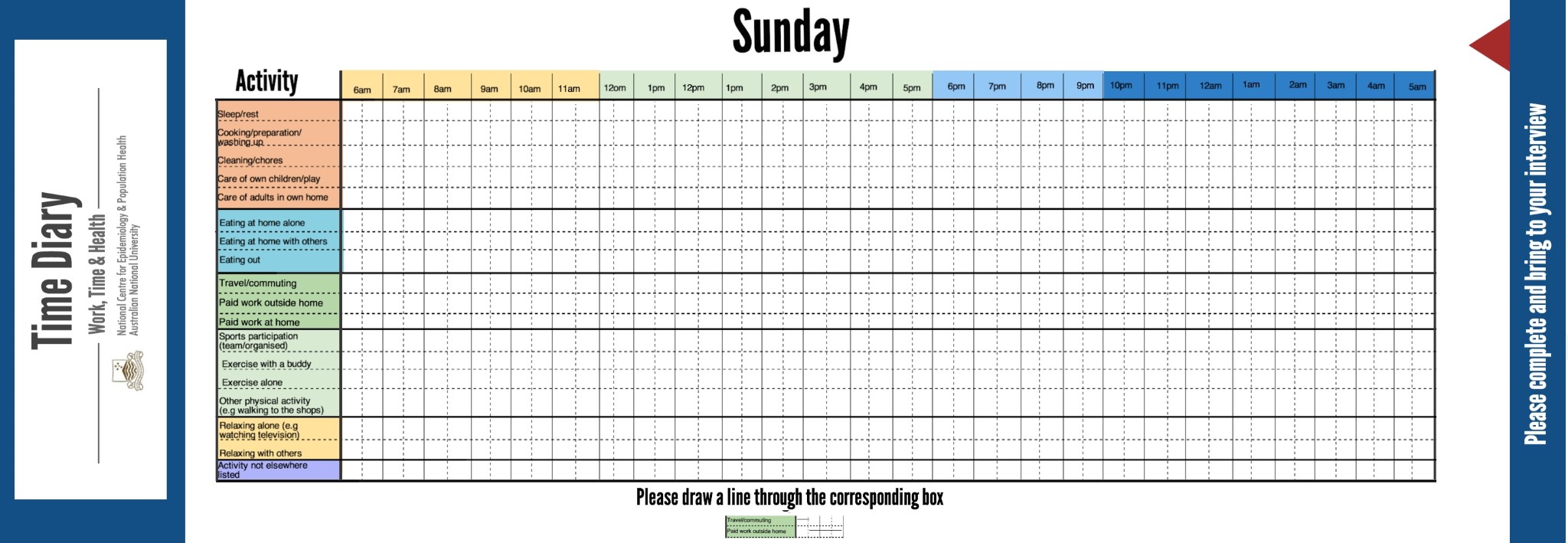

Supplement: S5 File — (DOCX) [file pone.0224542.s005.docx]

**S6 File. Work Time Control Protocol**


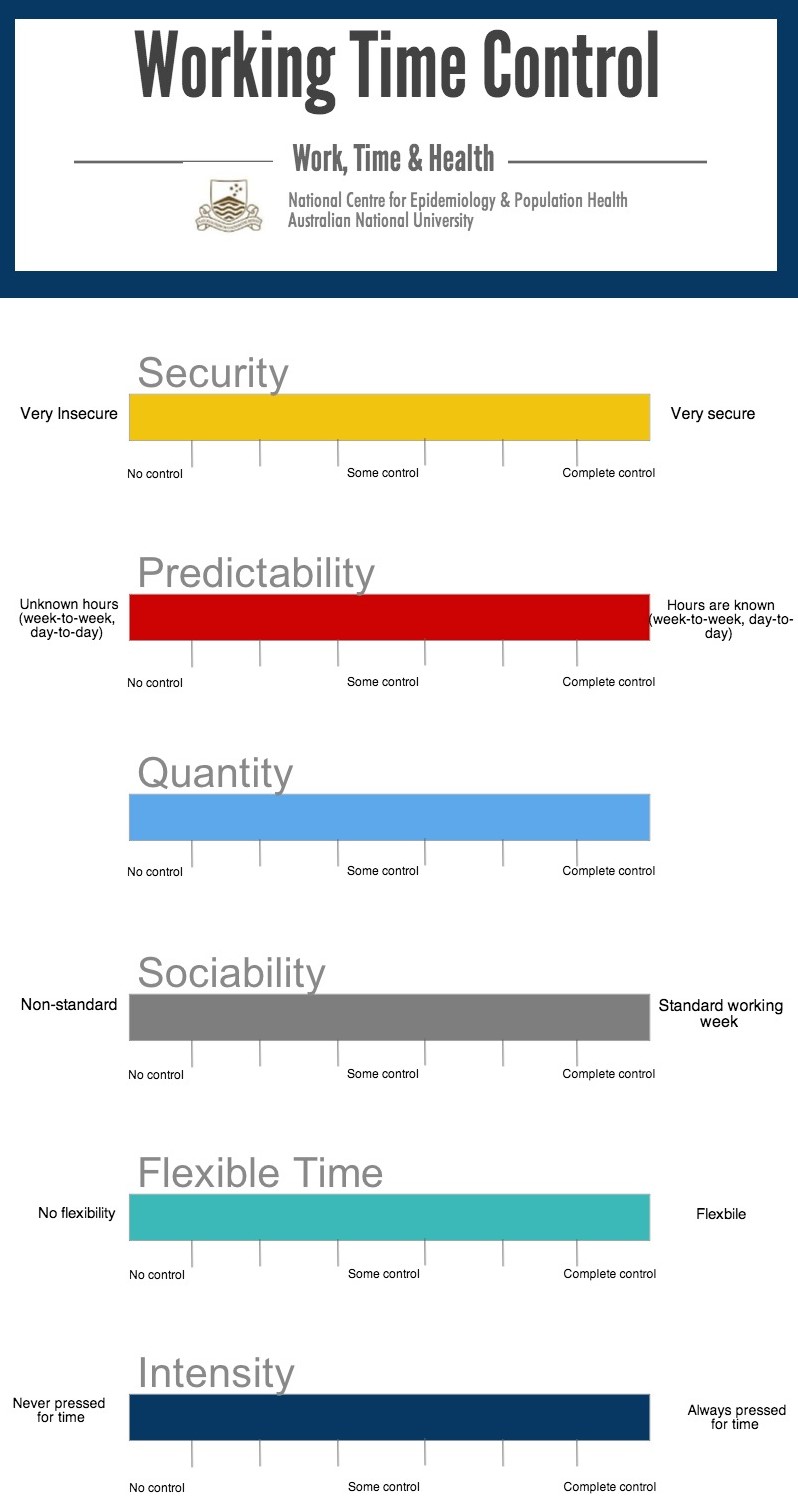

Supplement: S6 File — (DOCX) [file pone.0224542.s006.docx]
