# Supplementary material for: Flexible employment policies, temporal control and health promoting practices: A qualitative study in two Australian worksites
Source: PLoS One. 2019 Dec 20;14(12):e0224542. doi: 10.1371/journal.pone.0224542 (PMC6924681; doi:10.1371/journal.pone.0224542)
Supplement: S7 File — (DOCX) [file pone.0224542.s007.docx]

Work, Time & Health

ANU HREC protocol 2014/285

**S7 File. Interview Protocol**

1. Thank them for their interest and time
2. Ask about time constraints for the interview
3. Talk through information sheet
4. What to expect today: time, questions, recording
5. Any questions?
6. Participants to read and sign consent form
7. Ask socio-demographic survey questions
8. **Begin with time diary**
9. **Working times tools**
10. **Remainder of interview questions**
11. Ask permission to make contact if there are follow up questions
12. Provide voucher, with thanks

**Theme list**

Basic demographic information (occupation, age, family structure) (facilitated by a short oral survey)

The interaction between work and non-work activities: care and commuting specifically (if, when, how they interfere with one another)

Work time routines and 3 health practices; EATING, PHYSICAL ACTIVITY AND SLEEP – issue of trade-offs

Time pressure and self-rated health

Coping strategies for managing time

**Questions**

Working conditions scale & time diary

Can you tell me a little about the nature of your work (what you do)?

Discus time diary – how typical were these days?

Using working conditions scale:

- Do you work multiple jobs or just one?
- Do you feel that your job is secure?
- What type of hours do you work (how many, what times of the day)?
- Do you know what your hours will be day-to-day or week-to-week?
  - If not, how does this affect you/your family?
- When you’re at work, do you feel pressed for time? Can you explain how this feels? (e.g. anxious, busy)
  - If so, how do you manage this?
- There’s been a lot of discussion in the media and politics about ‘flexible work’. What does ‘flexible work mean to you?
  - Do you feel you can access flexible work arrangements?
  - If so, how does this help you manage other commitments or activities in your life?

**Flexible work arrangements**

Can you vary your start/finish times? Y/N

Can you vary when in the week you work? Y/N

Can you work reduced hours if you need? Y/N

Can you arrange your time to manage unexpected personal/family responsibilities? Y/N

Can you take extended leave? (Maternity? Study?) Y/N

Can you reduce work responsibilities if needed? Y/N

Can you work off site if needed? Y/N

Can you vary your employment contract (e.g. job share)? Y/N

Can you adjust your benefits? (e.g. buy extra annual leave) Y/N

How satisfied are you with your work?

How satisfied are you with your free time?

Work-life interference

Apart from work, do you have any other major commitments that take up your time?

- E.g. study, caring for children

Can you tell me about the last time work interfered with these activities (e.g. having to work extra hours)?

Can you tell me about the last time your partner’s work (if applicable) interfered with these other activities?

- Can you tell me a little about how you and your partner (or household) manage these time conflicts?
- Can you tell me about how you juggle work and other activities so that you can have time with family and friends? (e.g. do you work quicker, break domestic tasks up, multi-task?)
  - Is there anything that would make this easier?

Do you ever feel rushed or like you don’t have enough time outside of work?

- If so, when do you feel this way? How often?

Do you or your family have to skip any activities in order to fit work in?

If you have make a choice between a work commitment and another activity, how do you make this decision?

- Is there a recent example you could discuss where you had a conflict?

Health impacts

1. What activities do you do to keep fit and healthy or relax? Of these, are there any you’d like to do more of?
2. How do you fit this into your work schedule?
3. Can you please describe what you any exercise that you were doing last night (after 6pm)?

- What did you do? Were you alone or with others, where did you undertake the activity and at what time? How long approximately did the activity/leisure episodes take?

1. Can you tell me about your dinner last night?

- Where did you eat? What? Who did the food preparation? Were you alone or with others, where did you eat and at what time? How long approximately did the meal/eating episodes take?

1. Can I ask about this morning’s breakfast

- Where did you eat? What? Who did the food preparation? Were you alone or with others, where did you eat and at what time? How long approximately did the meal/eating episodes take?

1. Were these typical evenings in terms of eating and physical activity for a X day of the week (eg Tuesday)?
2. And once more: what about Sunday middle of the day/lunch time and any activity…. repeat the line of questioning as to their typicality/variation
3. Would you say that you have an established routine regarding your eating and exercisee?

- for breakfast. lunch, dinner and snacking?
- And physical activity routines?

1. Are you content with your answers to the above, or in an ideal world how would you change your patterns of eating and physical activity?

Prompts:

Are you able to plan home-cooked meals because your day is pretty predictable?

What about snacking and eating on the run? Are these common events because of hunger, lack of food in the house, your busy schedule? Are you able to share meals with your family and /or friends?

Can you exercise with some one? Stick to an exercise schedule? Is this a problem for you?

1. Does work ever interfere with cooking, exercising or other activities you do to keep healthy?

*Now turning to sleep?*

Do you normally sleep at night? Or does shift work mean that you sleep during the day?

How many hours of sleep did you get last night/yesterday? Was that typical of a X night/day?

Do you have a sleep routine across the week and the month?

If not, is this a problem? Is work a part of the problem, or is it more care duties etc?
