# Supplementary material for: Flexible employment policies, temporal control and health promoting practices: A qualitative study in two Australian worksites
Source: PLoS One. 2019 Dec 20;14(12):e0224542. doi: 10.1371/journal.pone.0224542 (PMC6924681; doi:10.1371/journal.pone.0224542)
Supplement: S8 File — (DOCX) [file pone.0224542.s008.docx]

**S8 File. Background Information + Job Time Strain**

Work, Time & Health

**ID NO. ………. DATE OF INTERVIEW………….. INTERVIEWER:……..**

**Age: Sex: M/F/Other**

**What country were you born in: Australia/…………..**

**Household/family structure**

**Marital states:**

**Children? Y/N No…… Ages? Living at home?**

**Non-parental care? Y/N Hrs/wk …………**

**Who do you live with? Mother/Father/Husband/Wife/Partner/Friends/Other**

**……………………………………………………………………………………………………………………..**

**……………………………………………………………………………………………………………………..**

**Highest level of education: ……………………………………………………………….....................................**

**Occupation: ……………………………………………………………………………..…………..…………….**

**Current position/role: ……………………………………………………………………..……………………...**

Refer to participant card 1. **ANZSCO Group: 1 2 3 4 5 6 7 8**

**Duties: …………………………………………………………………………………….……………………….**

**How long have you worked in this role? ………………………………………………………………………..**

**Employment**

**Status: full time/part time/ casual/ other……….**

Top of Form

Bottom of Form

**☐ Regular set hours each day/roster**

**☐ Flexible hours each day**

**☐ Regular set hours + extra hours**

**☐ Shift work, rotating roster**

**☐ Shift work, regular roster**

**☐ Set number of hours/roster, but days/time vary**

**☐ Hours vary day-to-day, week-to-week**

**Shift work:** day/night/both **Split shifts:** yes/no/other ……..

**How many shifts/days/hours do you work each week?**

**Days: M / T/ W/ TH/ F/ SA/ SU**

**Description:** (Day, night, weekend)……………………………………………

**Is there flexibility when you start or stop? Y/N**

**Who sets the hours you work? ……………………………………………….**

**Is there any difference between the number of hours you are paid/contracted for and the number of hours you actually work? Y/N**

**No. of hours on paper …………………. Actual total working hours ……………..**

**Membership in employee representative body? (e.g. union) …………………..**

Refer to participant card 2: **Taking everything into consideration, how satisfied to do you feel with your job?**

**Extremely dissatisfied 1 2 3 4 5 6 7 Extremely Satisfied**

**How often do you feel rushed or pressed for time?**

**Almost always / Often / Sometimes / Rarely / Never**

**In general, would you say your health is:**

**Excellent / Very good / Good / Fair / Poor**

Refer to participant card 3:

**Current gross household income level (ATO categories): A B C D E**

**Do you feel you can manage on this income? Yes/no ……………………………………………**

**I consider my workload reasonable** Yes/No

**I can complete my assigned workload during my regular working hours** Yes/No

**I have a say in decisions and actions that impact on my work** Yes/No

**I get adequate recognition from my immediate supervisor when I do a good job** Yes/No

**Does your workplace have a strong OH&S program?** Yes/No

**Does your job involve working to very tight deadlines?** Yes/No

On a scale of importance from 1 to 10:

**How important is it to you that your work provides opportunities for you to maintain or improve your health?**

Not at all important 1 2 3 4 5 6 7 8 9 10 Most important thing

**Participant card** 1

| **ASCO**  **Code** | **ASCO Group and scope** |
| --- | --- |
| **1** | **Managers and Administrators:**  Magistrate, general managers, company secretaries, manufacturers, policy and planning managers, sales and marketing managers, farmers and farm managers |
| **2** | **Professionals:**  Natural and physical scientists, accountants, engineers, marketing and advertising professionals, school teachers, social welfare & legal professionals, computing professionals |
| **3** | **Associate Professionals:**  Technical officers, shop manager, real estate associates, branch accountants and managers, enrolled b=nurses, ambulance officers, massage therapists, sportspersons, police officers |
| **4** | **Tradespersons and Related Workers:**  Mechanical engineering trades, toolmaker, motor mechanics, electricians, carpenters, signwriters, bakers and meat trades, farm overseers, gardeners, hairdressers, florists |
| **5** | **Advanced Clerical and Service Workers:**  Secretaries and personal assistants, credit officers, travel attendants, bookkeepers, |
| **6** | **Intermediate Clerical, Sales and Service Workers:**  General clerks. Receptionists, library assistant, child care workers, travel agents, prison officers, hospitality workers |
| **7** | Intermediate Production and Transport Workers:  Forklift drivers, engine and boiler operators, sewing machinists, chemical production machine operator, truck driver, miners, storeperson, seafarer |
| **8** | Elementary Clerical, Sales and Service Workers:  Mail sorter, betting clerk, cashier, telemarketer, housekeeper, security officer, sales demonstrators, sales assistants |
| **9** | **Labourers and Related Workers:**  Cleaners, hand packers, product assemblers, handypersons, fast food cooks, nursery and garden labourers, railway labourers, plumbers assistant |

**Time, Work and Health**

**Participant card 2**

**Which best describes you gross (before tax) household income level per year?**

**A Under $20 000**

**B $21000 - $40 000**

**C $41 000 - $70 000**

**D $71 000 - $104 000**

**E $105-000- $130 000**

**F $131 000 - $170 000**

**G >$170,000**
