# Supplementary material for: Flexible employment policies, temporal control and health promoting practices: A qualitative study in two Australian worksites
Source: PLoS One. 2019 Dec 20;14(12):e0224542. doi: 10.1371/journal.pone.0224542 (PMC6924681; doi:10.1371/journal.pone.0224542)
Supplement: Attachment — Submitted filename: Response to Reviewers_PONE-D-19-16021.docx [file pone.0224542.s009.docx]

12 September, 2019

**Response to Reviewers: PONE–D-19-16021**

Dear Dr. Loerbroks,

Please find our point-by-point response to the matters raised by you and the 2 reviewers. We thank you all for valuable remarks and believe that we have strengthened the paper considerably as a result.

1. *Please ensure that your manuscript meets PLOS ONE's style requirements*

We have attended to the format instructions, but note that we need assistance with formatting of Tables 1 and 2.

2. *Please include additional information regarding the survey or questionnaire used in the study and ensure that you have provided sufficient details that others could replicate the analyses*.

Pages 9-10 of the manuscript contains reference to the 5 time tool instruments and questionnaires that study participants responded to. Each has been uploaded as Appendices 4-8.

3. *We note that you have indicated that data from this study are available upon request. PLOS only allows data to be available upon request if there are legal or ethical restrictions on sharing data publicly.*

We are reluctant to release the interview transcripts into the public domain because there is a high likelihood given the small and select sample (N=28) that someone might be able to identify the respondents. Both work sites are located in regional towns and are small: one has 60 employees and the other 200. The transcripts contain respondents’ ages, education, gender, organisational role, family characteristics and other personal data. Given the descriptions that they provide of their workplaces within the transcripts, alongside the foregoing information, it would be relatively easy to identify each workplace and each respondent despite their de-identification. Furthermore as highlighted in our letter to you, we did not ask the participants for permission to share their transcripts.

*Please also provide contact information for a data access committee, ethics committee, or other institutional body to which data requests may be sent.*

The study was approved by the Australian National University (ANU) Science and Medical Delegated Ethics Research Committee in 2014 (Approval 2014/285). ANU researchers are bound by the Australian Government’s Code for the Responsible Conduct of Research <https://nhmrc.gov.au/research-policy/research-integrity/release-2018-australian-code-responsible-conductresearch>.

An annual progress report of the research has been undertaken between 2014-2017 (when it was completed) in line with ANU Human Ethics procedures. The appropriate contact for this committee, now called Humanities & Social Sciences Delegated Ethics Review Committee, is: [human.ethics.officer@anu.edu.au](mailto:human.ethics.officer@anu.edu.au). See link to website

<https://services.anu.edu.au/planning-governance/governance/humanities-social-sciences-delegated-ethics-review-committee>

4. We have provided an amended Funding Statement that declares *all* the funding or sources of support received during this specific study (whether external or internal to your organization).

a. *Please state what role the funders took in the study.  If any authors received a salary from any of your funders, please state which authors and which funder. If the funders had no role, please state:*

The study was funded by the Australian Research Council Discovery Programme grant number DP 140102856. The funders had no role in study design, data collection and analysis, decision to publish, or preparation of the manuscript.

5. *We note that your paper includes detailed descriptions of individual patients/participants… please provide a consent form following the PLOS guidelines*.

All participants signed a Consent Form (uploaded as Appendix 3 alongside the Letter of Invitation-Information sent to employers and employees describing the study as Appendix 1 and 2) as required by the ANU Science and Medical Ethics Research Committee. We used the prescribed Committee form. All consent forms are kept in a secure location within the Research School of Population Health at the University, along with the transcripts and time tool forms completed by each participant. These materials will be kept for 5 years after the completion of the research (2017). It is now not realistic to return 4 years later to the 2 worksites and find the 28 individuals for the purposes of them signing the PLOS consent form. Moreover, it is perhaps unethical as they did not consent to be recontacted.

6. *Please consider changing the title*

The title has been changed from ‘’Industrial relations transformed: temporal control and preventive health practices’’

To “Flexible employment policies, temporal control and health promoting practices: A qualitative study in two Australian worksites”

*Data availability: Please list a contact person or committee which can grant access to the data other than the corresponding author*

Please see above under point 3.

*Methods: was the sample size determined a priori or is it the result of data collection until saturation?*

Lines 201-203 now read: We aimed to recruit a non-representative sample of around about 12 participants at each worksite based on research showing that new relevant information is rarely gained through additional interviews [45}

*Did you use a topic guide? if so, please provide it*

Please see Appendix 8, now uploaded.

- Line 196-198: How does a presentation of findings contribute to validation? Did you discuss findings with the audience?

Lines 228-232 now read: To validate the team’s interpretations, participating organisations received a summary of findings for their worksite and were provided with an oral presentation at a specially convened staff meeting. At the presentation, those interviewed and other interested staff could seek clarification or modification of interpretations. There was one substantial instance of the latter at the construction worksite.

- *The companies employed 60 and 200 workers, respectively. How exactly were participants selected from those samples? How many of the 28 individuals participated per company?*

The participants self-selected on the basis of management drawing attention to a flyer requesting volunteers sent to the workplaces by the study team. We sought a diverse range of employees and approximately 12 in each site. We were fortunate that 15 out of 200 employees in the larger worksite and 13 out of 60 in the small site responded. Table 1 on pages 11-12 sets out the characteristics of the 2 samples, and it indicates a range of job types, years with the employer and other indicators of a diverse pool of respondents in line with the methodological approach.

*Results: do you have any additional descriptive data related to the participants (e.g. their age and type of contract, e.g. full time v spart time)?. Give your research questions it seems particularly interesting to what extent findings/themes differed among those with full-time vs part-time employment*

Table 1 contains the requested information. Only 2 of the 28 participants were part-time making any thematic differences with full-time workers elusive. We have however now included a new section (pages 25-28) entitled ‘’Common finding: Need for support from partners and extended family’’ which contains material as to why women may seek out part-time work.

*… reflect in detail on how your methodological approach may have affected the nature of your findings (e.g. limitations); for instance, participants completed a diary on a single working day: to what extent can you be certain that this was a typical working day? One may suspect that dairies can only be completed on working days with higher than usual job control. Could this have affected the subsequent interviewing and thus the range of observations?*

We have included a new section Strengths and limitations (lines 739-759) at the end of the Discussion where we reflect on our methodological approach. As to the diary, participants were asked to complete one for a working day and one for a Sunday prior to, but close to, the interview. In the interviews, participants were asked how typical the entries were. They were very open about any discrepancies from a ‘normal’ day.

*Reviewer #1: The only Thing that could be improved in my view is the theoretical debate the paper is pursuing.*

We have written a more fulsome Conclusion (an additional 470 words) to include discussion of the theoretical-research directions that we see as flowing from the study (see lines 781-831). Specifically, we propose that Moen’s conceptualisation is inadequate and that all studies of work demand-control have to consider the wider employment policy environment and societal conditions in which organisational-level dynamics are being operationalised. We note that the ongoing relevance of classic labour studies concerns.

Reviewer #2

*the broader discussion regarding the development of “real” decision latitude in relation to formal structures and tightening markets is much older than the authors seem to be aware of*

The original manuscript does contain a reference from 1989. However, we have now included 3 new references alongside the statement: “Job control insights have provoked decades of research activity and management practices [15,16,17,18]”. Two of these refer to Scandanavian research, as pointed to by the reviewer, as does reference 19.

*… more words of [methodological] caution should be issued*

Please refer to the new section on Study strengths and limitations

*Health behavior is much more and it brings up the question whether the interviewers have had a narrow focus in their interviewing about health behavior*.

We are well aware of this point, but we intentionally chose to narrow the health risk factors to eating and physical activity for 2 reasons: they are major risk factors for obesity and weight related non-communicable disease and this is our area of expertise having written 2 books and numerous articles on the social determinants of obesity. Refer to lines 133-159 for a thorough review of the studies which focus on the possible links between flexible work and eating and physical activity. We did ask about sleep, as some research shows a link to weight, but did not include that analysis within the paper due to length blow-out. Some reference is made in the article to wanting more sleep as well as more leisure time.

*the discussion is interesting and brings up the important point that use of time must be analysed in a total context and that what may seem on surface as a good solution is not always so good.*

We are pleased with this view, and have added some concluding remarks regarding employment policy directions on the basis of recent reports from EU bodies and the International Labour Organisation (refs 23, 60-62). The abstract also now has an additional paragraph reflecting this content.

I am happy to follow up with any questions,

Sincerely

Jane Dixon, PhD
